# Acute effects of cardiac contractility modulation on human induced pluripotent stem cell–derived cardiomyocytes

**DOI:** 10.14814/phy2.15085

**Published:** 2021-11-03

**Authors:** Tromondae K. Feaster, Maura Casciola, Akshay Narkar, Ksenia Blinova

**Affiliations:** ^1^ Office of Science and Engineering Laboratories Center for Devices and Radiological Health US Food and Drug Administration Silver Spring Maryland USA

**Keywords:** calcium handling, cardiac contractility modulation, cardiomyocytes, hiPSC‐CM

## Abstract

Cardiac contractility modulation (CCM) is an intracardiac therapy whereby nonexcitatory electrical simulations are delivered during the absolute refractory period of the cardiac cycle. We previously evaluated the effects of CCM in isolated adult rabbit ventricular cardiomyocytes and found a transient increase in calcium and contractility. In the present study, we sought to extend these results to human cardiomyocytes using human induced pluripotent stem cell–derived cardiomyocytes (hiPSC‐CMs) to develop a robust model to evaluate CCM in vitro. HiPSC‐CMs (iCell Cardiomyocytes^2^, Fujifilm Cellular Dynamic, Inc.) were studied in monolayer format plated on flexible substrate. Contractility, calcium handling, and electrophysiology were evaluated by fluorescence‐ and video‐based analysis (CellOPTIQ, Clyde Biosciences). CCM pulses were applied using an A‐M Systems 4100 pulse generator. Robust hiPSC‐CMs response was observed at 14 V/cm (64 mA) for pacing and 28 V/cm (128 mA, phase amplitude) for CCM. Under these conditions, hiPSC‐CMs displayed enhanced contractile properties including increased contraction amplitude and faster contraction kinetics. Likewise, calcium transient amplitude increased, and calcium kinetics were faster. Furthermore, electrophysiological properties were altered resulting in shortened action potential duration (APD). The observed effects subsided when the CCM stimulation was stopped. CCM‐induced increase in hiPSC‐CMs contractility was significantly more pronounced when extracellular calcium concentration was lowered from 2 mM to 0.5 mM. This study provides a comprehensive characterization of CCM effects on hiPSC‐CMs. These data represent the first study of CCM in hiPSC‐CMs and provide an in vitro model to assess physiologically relevant mechanisms and evaluate safety and effectiveness of future cardiac electrophysiology medical devices.

## Graphical abstract

hiPSC‐CMs’ contractility increases in response to biphasic nonexcitatory electrical pulses delivered during refractory period mimicking clinical CCM device therapy. CCM‐induced inotropic effects to be in part mediated by β‐adrenergic signaling and discovered that CCM pulses have modulated myofilament calcium sensitivity and L‐type calcium channels in hiPSC‐CMs. CCM‐induced kinetic enhancements remained intact when the positive inotropy was blunted suggesting that CCM may provide a lusitropic benefit to patients independent of the inotropic benefit.

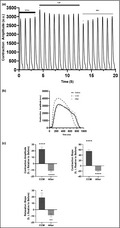

## 1 INTRODUCTION

Cardiac contractility modulation (CCM) is a cardiac therapy whereby nonexcitatory electrical pulses are delivered to the heart during the absolute refractory period of the cardiac cycle (Campbell et al., 2020). Recently, the first CCM device was approved in the United States to treat patients with heart failure (HF) (NYHA III), that are in normal sinus rhythm, not indicated for cardiac resynchronization therapy (CRT), remain symptomatic despite guideline directed medical therapy and have a left ventricular ejection fraction ranging from 25 to 45% (Campbell et al., 2020; FDA.gov, 2019; SSED, 2019). In the future, novel CCM devices are expected to be developed to address additional patient populations and device functionalities. The CCM device is implanted in the pectoral region and contact leads are placed in the myocardium. Novel cardiac electrophysiology medical devices, including CCM and CRT have been developed to treat patients with drug‐resistant HF. Although CRT is the first‐line treatment for patients with HF displaying low ejection fraction (< 35%), abnormal electrical activity, and prolonged QRS duration (Jaffe & Morin, 2014), there still remains a significant population of HF patients (e.g., 60–70%) with normal electrical activity or QRS duration. CCM may be useful for such patients who may not be eligible for CRT. Consequently, there is a significant gap for viable treatment option for certain HF populations and CCM is heralded as a potential solution.

Lack of human in vitro models to evaluate cardiac medical device safety and effectiveness currently hinders the regulatory review process and results in the significant burden on animal models (Harris et al., 2013). Thus, the direct effects of CCM stimulation on human cardiomyocyte physiology remains poorly understood. Although preclinical models have provided potential mechanistic insight into our understanding of CCM, a major hinderance to the detailed study of CCM biology has been the lack of appropriate in vitro human cardiac models (Strauss & Blinova, 2017). In principle, human induced pluripotent stem cell–derived cardiomyocytes (hiPSC‐CMs) may be a useful model to assess the molecular and functional effects of CCM in human cardiac tissue in vitro. In this work we demonstrate that hiPSC‐CMs respond to acute electrical stimulation mimicking clinical CCM stimulation by transient increase in contractility. This work, the first hiPSC‐CM CCM device study, elucidates the acute effects of CCM on human cardiomyocyte biology and provides important insights and evidence of CCM mechanisms. Here, we establish a standardized hiPSC‐CM‐based method to quantify acute CCM effects in vitro.

## 2 METHODS

### 2.1 human induced pluripotent stem cell–derived cardiomyocyte derivation and culture

Cryopreserved hiPSC‐CMs (iCell Cardiomyocytes^2^, 01434, Fujifilm Cellular Dynamic, Inc.) were thawed and plated according to the manufacturer's instruction. All hiPSC‐CMs were derived from the same hiPSC line, which was reprogrammed from fibroblast donor tissue, isolated from an apparently healthy normal Caucasian female, <18 years old (Ma et al., 2011). Briefly, 750,000 viable cells were plated per well of a 6‐well plate on 0.1% gelatin and allowed to recover from cryopreservation for at least 2 days at 37°C. Cells were then dissociated, each well was washed twice with 2X volume (i.e., 4 ml) of Dulbecco's Phosphate‐Buffered Saline (DPBS) then 1 ml of TrypLE™ Express was added and cells we incubated for 15 min at 37°C to dissociate. M3 medium consisting of RPMI 1640 with glucose (Invitrogen, cat# 11875); 2% B27 with insulin (Invitrogen, cat#17504‐044); 1% Pen‐Strep (Invitrogen, cat#17504) (Feaster et al., 2015) was used to quench the TrypLE™ Express and cells were collected in a 15‐ml conical tube and centrifuged at 200 g for 5 minutes at 25°C. Cells were then resuspended in a small volume of M3 (e.g., 2 ml), filtered with a 100 µm filter, counted, and plated on Matrigel mattress substrate as previously described (Feaster et al., 2015). Matrigel mattresses were arrayed horizontally in 48‐well glass bottom plates, 1 mattress (i.e., ~1 µl) per well (Figure S1c) and allowed to incubate for 8–10 min at which point 15,000 viable cells in 100 µl volume were added directly to the Matrigel mattress. After 10–15 min incubation at room temperature, the volume was adjusted to 300 µl per well. M3 medium was changed every day thereafter and cells well allowed 2–5 days before experiments were performed (Supplemental Video, of contracting hiPSC‐CMs on Matrigel mattress).

### 2.2 Electrical field (CCM) stimulation

HiPSC‐CMs were stimulated with a commercial pulse generator and software AM‐Systems (Model 4100, World Precision Instruments, Sarasota, FL) in conjunction with the CellOPTIQ Platform (CellOPTIQ, Clyde Biosciences) (Figure S1d). The removable platinum wire electrodes (interelectrode distance 2.0 mm, and width 1.0 mm) (Figure S3) were compatible with standard 48‐well glass bottom plates (MatTek), electrodes were placed in each well sequentially (Figure S1d, right panel). Both pacing and CCM electrical pulses were delivered through the same pair of electrodes, resulting in field stimulation of the hiPSC‐CMs as previously described in (Blinova et al., 2014). Square wave electrical pacing pulses (i.e., monophasic) were generated and hiPSC‐CMs were paced at 1 Hz (2 ms stimulus pulse duration), 14 V/cm (64 mA). CCM stimulation was delivered as four biphasic pulses of 5.14 ms pulse duration (20.56 ms total duration), 28 V/cm (128 mA, phase amplitude), zero interphase interval. The delay between pacing pulses and CCM stimulation was 30 ms. CCM pulse parameters were comparable with the setting typically used in clinical practice (Figure S1d, right panel) (Campbell et al., 2020). Varying CCM pulse amplitudes were tested to determine the optimal CCM experimental amplitude of 10 V (i.e., 28 V/cm; 128 mA, phase amplitude). (Figure S4a,b) to enable robust CCM response in hiPSC‐CMs.

### 2.3 Measurement of contractile properties

Video‐based Contractility Platform and Software (CellOPTIQ, Clyde Biosciences), based on pixel displacement, was used to measure hiPSC‐CM contractility (Saleem et al., 2020). Briefly, hiPSC‐CM were plated on Matrigel Mattress substrate in 48‐well glass bottom plates. hiPSC‐CMs were imaged directly in plates by an inverted fluorescence microscope (Zeiss) using 40× objective. A camera connected to the front port of the microscope was used for contraction acquisition. Temperature, 37°C, and 5% CO_2_ were maintained by environmental control chamber (OKOLAB INC.). Experiments were performed in 300 µl of Tyrode's solution containing (in mmol/L): CaCl_2_ 0.5, NaCl 134, KCl 5.4, MgCl_2_ 1, glucose 10, and HEPES 10, pH adjusted to 7.4 with NaOH. Contractile properties including contraction amplitude, contraction slope, relaxation slope, time to peak 50%, time to peak 90%, time to baseline 50%, time to baseline 90%, contraction duration 10%, contraction duration 50%, and contraction duration 90% were evaluated. Each monolayer was evaluated to find an area of uniformity, this region of interest (ROI) was selected near the center of the Matrigel mattress monolayer and kept constant throughout the experiment. For each experimental group before CCM, during CCM, and after CCM a minimum 4‐second recording was taken and analyzed per group as previously described.

### 2.4 Measurement of intracellular calcium

HiPSC‐CMs were loaded with calcium‐sensitive probe (10 µM Fluo‐4F AM, Molecular Probes, F14201) prepared in Tyrode's solution containing (in mmol/L): CaCl_2_ 0.5, NaCl 134, KCl 5.4, MgCl_2_ 1, glucose 10, and HEPES 10, pH adjusted to 7.4 with NaOH with the addition of Pluronic F‐127 for 25 min at room temperature (Blinova et al., 2014) and imaged directly in 48‐well glass bottom plates (MatTek) by an inverted fluorescence microscope (Zeiss). The camera attached to the side port of the microscope was used to position the hiPSC‐CMs for fluorescence‐based calcium transient measurement using 40× objective (Lamore et al., 2017). Calcium‐transient acquisition and analysis was conducted using CellOPTIQ (Clyde Biosciences, Glasgow, UK) hardware and software. All data were collected at 37°C and 5% CO_2_. Photomultiplier tubes were used to record change in fluorescence collected at 470 nm during CCM stimulation compared with before CCM control. Calcium handling parameters including calcium‐transient amplitude (CaT), calcium‐transient slope (Up), calcium‐transient slope (down), time to peak, calcium‐transient rise time, calcium‐transient duration 50%, and calcium‐transient duration 90% were evaluated. For each monolayer, an ROI was selected near the center of the Matrigel mattress monolayer and kept constant throughout the experiment. For each experimental group before CCM, during CCM, and after CCM, a minimum 4‐second recording was taken and analyzed per group as described above.

### 2.5 Action potential recordings

Cardiac action potentials were measured from hiPSC‐CM monolayer with voltage‐sensitive probe. Briefly, using the same set up as above hiPSC‐CM were incubated with [20 µM] di‐4‐ANEPPS (Life Technologies) prepared in Tyrode's solution containing (in mmol/L): CaCl_2_ 0.5, NaCl 134, KCl 5.4, MgCl_2_ 1, glucose 10, and HEPES 10, pH adjusted to 7.4 with NaOH with the addition of Pluronic F‐127, ensuring that the exposure time to di‐4‐ANEPPS was 2 min as in (Blinova et al., 2014). The cells were allowed to recover from staining before APD data were collected. Optical assaying of action potential was conducted using CellOPTIQ (Clyde Biosciences, Glasgow, UK) hardware and software (Blinova et al., 2019) using 40× objective. HiPSC‐CMs were excited at 470 nm and motion artifacts were minimized by using fluorescent dyes in ratiometric mode. The two emission wavelengths used in ratiometry were 510–560 nm and 590–650 nm. All data were collected at 37°C and 5% CO_2_. For each monolayer, an ROI was selected near the center of the Matrigel mattress monolayer and kept constant throughout the experiment. Action potential parameters including action potential rise time (TRise), action potential duration 50% (APD 50), action potential duration 75% (APD 75), and action potential duration 90%, (ADP 90) were evaluated. For each experimental group before CCM, during CCM, and after CCM, a minimum 4‐s recording was taken and analyzed per group as described above.

### 2.6 Compounds and pharmacological assay

All compounds were resuspended based on manufacturers’ recommendations; metoprolol (Sigma, M5391‐10G) solution was prepared using sterile water. Each experiment typically measured pharmacological response for 6 to 9 wells taken from independent cell thawing and plating. HiPSC‐CMs were incubated with Tyrode's solution containing (in mmol/L): CaCl_2_ 0.5, NaCl 134, KCl 5.4, MgCl_2_ 1, glucose 10, and HEPES 10, pH adjusted to 7.4 with NaOH with vehicle or drug and allowed to equilibrate for approximately 5 minutes before experiments. Pharmacological assays were performed with external calcium concentration [Ca]_o_ range 0.25–2 mM Tyrode solution (Feaster et al., 2015).

### 2.7 Numerical electric field modeling

Finite element analysis software Sim4Life Version 6.2 (ZMT Zürich MedTech AG, Zürich, Switzerland) was used to solve in quasi‐static conditions the electric (E) field distributions within the cell monolayer during CCM pulse delivery. A 3D geometry was constructed with dimensions equivalent to those in the experimental setup. Each treatment was performed in a single well of a 48‐well glass bottom plate with a thickness of 1.39 mm and radius of 5.19 mm. The platinum electrodes (8 mm length, 1.04 mm diameter, 2 mm interelectrode distance) were positioned in each well. The electrodes were immersed in a 2.2 mm thick layer of material mimicking the dielectric properties of the Tyrode solution used during the experiments (conductivity 3.1 S/m at 37C). A finer mesh was used that consisted of 1 M elements (Figure S3) shows the intensity of the E field in the plane perpendicular to the electrodes when 1 V is applied. In the ROI where the experimental measurements are taken, the intensity of the E field is homogenous reaching a value of approximately 2.8 ± 0.3 V/cm for 1 V applied (Figure S3).

### 2.8 Statistical analysis

All statistical analyses were performed using GraphPad Prism 8 software (Prism 8, GraphPad Software, CA). For evaluation of immediate effects (i.e., last before beat, first CCM beat, and first after beat). The featured beat from each group were combined and averaged. For evaluation of all beats, all beats in each group (i.e., before, CCM, and after) were combined and averaged. Differences among the groups are presented as mean ±standard error of the mean (SEM). Differences were assessed as percent change relative to before CCM using one sample *t*‐test, hypothetical values of zero. Results were considered statistically significant if the *p*‐value was less than 0.05.

## 3 RESULTS

### 3.1 Human in vitro cardiac contractility modulation model

Commercially available hiPSC‐CMs were evaluated in monolayer format on flexible substrate (i.e., Matrigel mattress) (Feaster et al., 2015; Wang et al., 2018) (Figure S1a–c; Video S1) and submaximal extracellular calcium concentration [Ca]_o_ [0.5 mM] comparable with the EC_50_ value (Mannhardt et al., 2016, 2017). At higher calcium concentrations (e.g., 2 mM), we observed blunted CCM‐induced inotropic effect (Figure S2) as such the work described here will focus predominately on submaximal extracellular calcium concentrations. The acute effects of standard clinical CCM pulse parameters (Figure S1d, right panel) on human cardiac physiology (i.e., contractility, calcium handling, and electrophysiology) were quantified.

### 3.2 Cardiac contractility modulation increases contractility of hiPSC‐CMs

**FIGURE 1:**
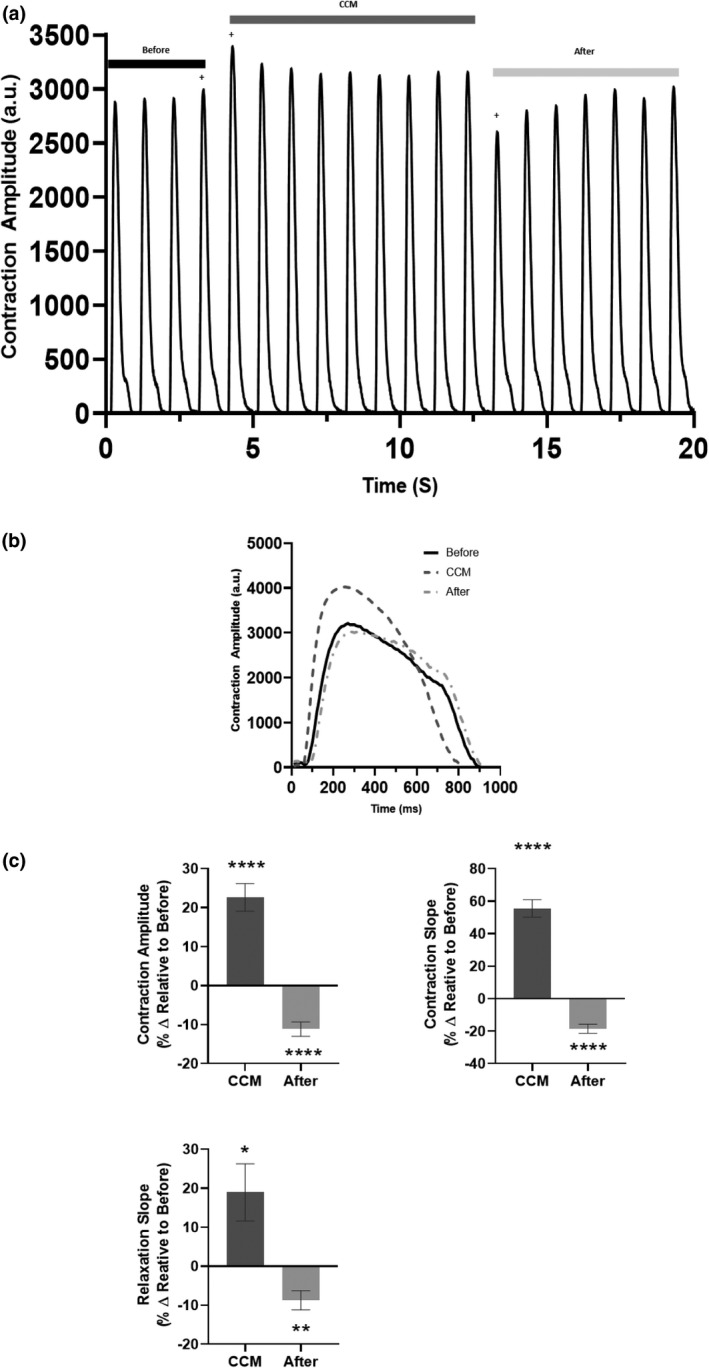
Acute effects of cardiac contractility modulation (CCM) on human induced pluripotent stem cell–derived cardiomyocytes’ contractile properties. (a) Representative contraction recording for before (5V), CCM (10V), and after (5V). (b) Representative contraction traces of immediate effects (i.e., last before beat, first CCM beat, and first after beat, indicated by plus). (c) Summary bar graphs of immediate effects. Percent change, data are mean ±SEM. *n* = 23. *p < 0.05, **p < 0.01, ***p < 0.001, ****p < 0.0001

HiPSC‐CMs exposed to clinically relevant CCM pulse parameters (Figure S1d, right panel) (Campbell et al., 2020) exhibited measurable differences in cardiac contractility that subsided immediately when the CCM signal was eliminated. For the first CCM beat, there was a significant increase in contraction amplitude (Figure 1a,b). Additionally, we observed faster contraction kinetics (contraction slope) and relaxation kinetics (relaxation slope) (Figure 1c). These effects remained for the entire duration of CCM stimulation (Table S1). These results demonstrate that acute clinical CCM stimulation enhance hiPSC‐CMs contractile properties in an in vitro human cardiomyocyte model.

### 3.3 Cardiac contractility modulation modifies calcium handling properties of hiPSC‐CMs

**FIGURE 2:**
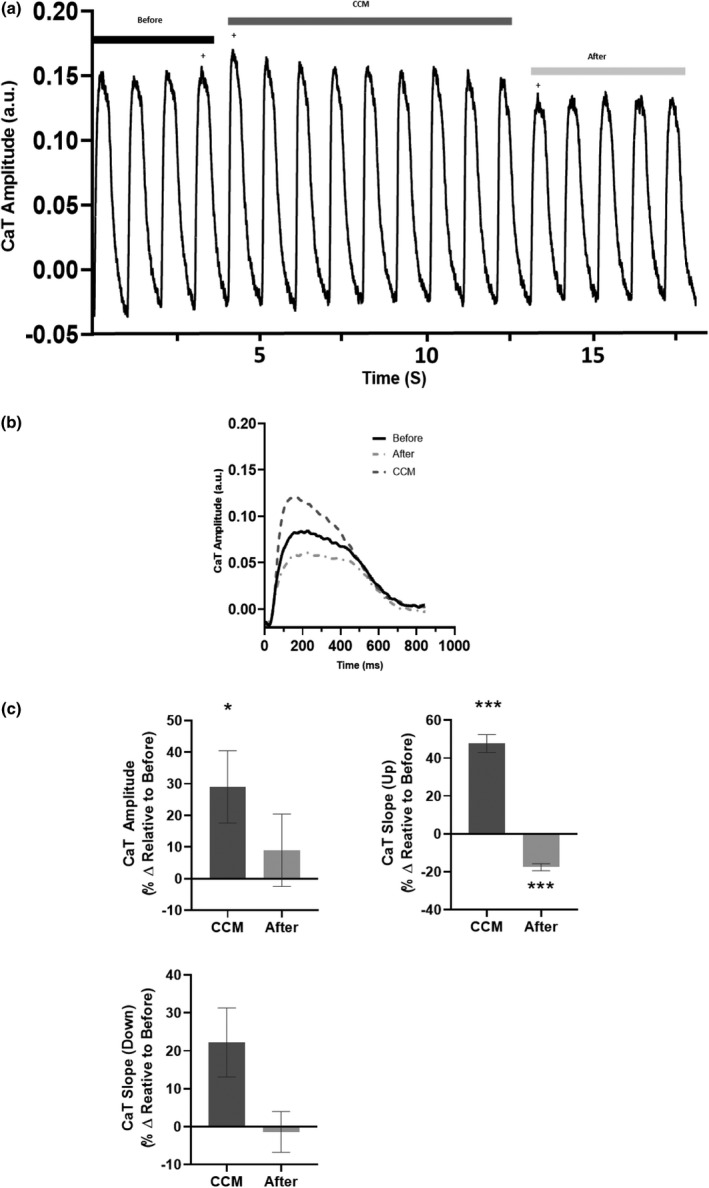
Acute effects of cardiac contractility modulation (CCM) on human induced pluripotent stem cell–derived cardiomyocytes’ calcium handling properties. (a) Representative calcium recording for before (5V), CCM (10V), and after (5V). (b) Representative calcium transients of immediate effects (i.e., last before beat, first CCM beat, and first after beat, indicated by plus). (c) Summary bar graphs of immediate effects. Percent change, data are mean ±SEM. *n* = 5–13. *p < 0.05, **p < 0.01, ***p < 0.001, ****p < 0.0001

Next, we evaluated the effects of CCM stimulation on intracellular calcium handling properties. HiPSC‐CM displayed modified calcium handling properties that subsided when the CCM signal was eliminated (Figure 2). On the first CCM beat, the intracellular calcium‐transient amplitude was significantly increased (Figure 2a,b) relative to before CCM. Moreover, CCM stimulation immediately resulted in faster calcium handling kinetics (CaT Slope, Up) (Figure 2c). Additionally, the effects of CCM on intracellular calcium handling remained for the entire duration (Table S2) of CCM stimulation and were eliminated when the CCM signal was eliminated. These data demonstrate acute CCM stimulation, with standard clinical CCM pulse parameters, modifies hiPSC‐CM intracellular calcium handling properties in vitro.

### 3.4 Cardiac contractility modulation shortens action potential duration in hiPSC‐CMs

**FIGURE 3:**
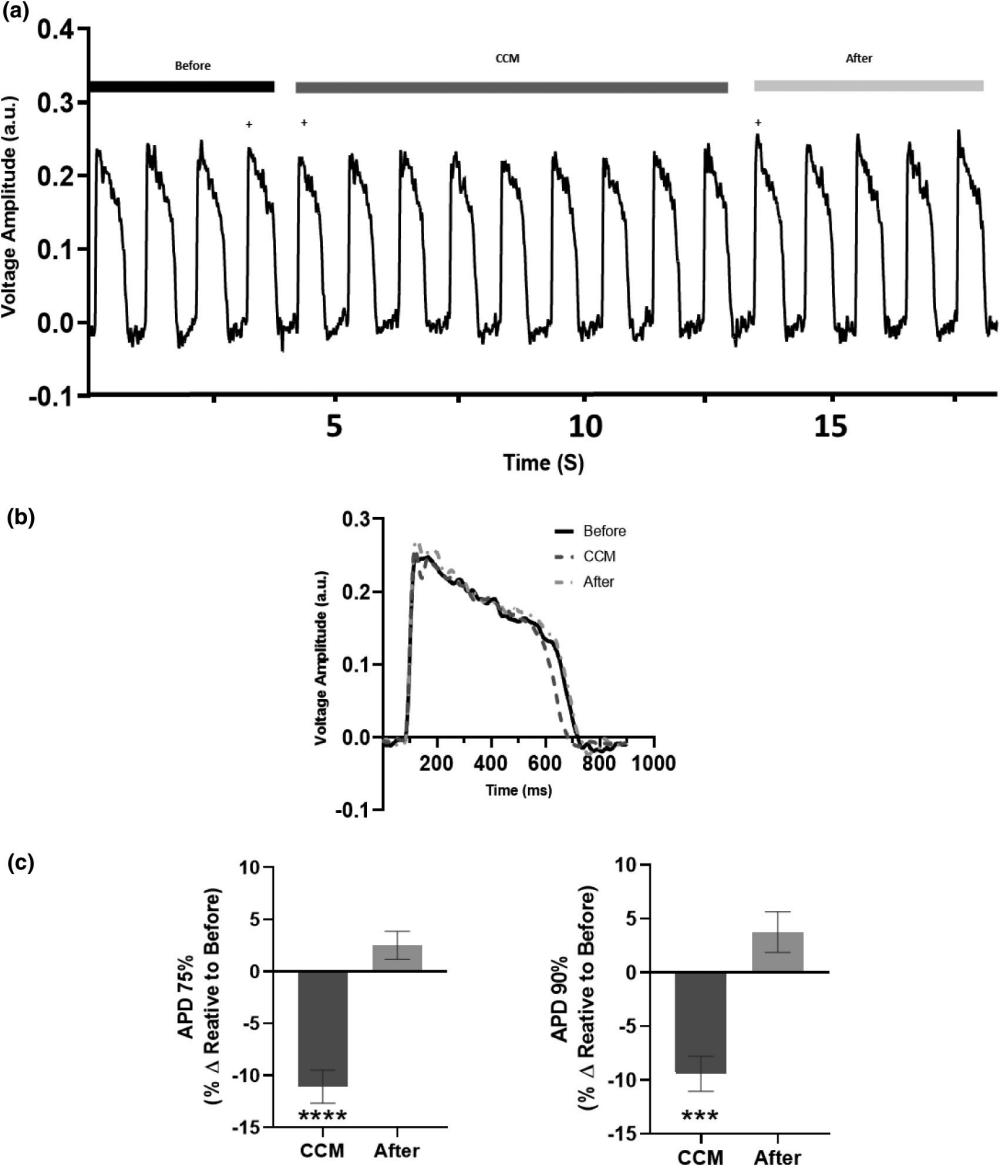
Acute effects of cardiac contractility modulation (CCM) on human induced pluripotent stem cell–derived cardiomyocytes’ electrophysiology. (a) Representative action potential recording for before (5V), CCM (10V), and after (5V). (b) Representative action potential morphology of immediate effects (i.e., last before beat, first CCM beat, and first after beat, indicated by plus). (c) Summary bar graphs of immediate effects. Percent change, data are mean ±SEM. *n* = 12. *p < 0.05, **p < 0.01, ***p < 0.001, ****p < 0.0001

To assess the acute effects of clinical CCM parameters on human cardiomyocyte electrophysiology, we evaluated hiPSC‐CM action potentials. Given that CCM is delivered to the myocardium during the absolute refractory period, it is thought to have negligible effects on electrophysiological properties, as the heart is unable to achieve depolarization. CCM stimulation immediately altered electrophysiological properties on the first CCM beat by significantly shortening APD (Figure 3). Likewise, these effects remained for the entire duration (Table S3) of CCM stimulation and were eliminated when the CCM signal was eliminated.

### 3.5 Effect of β‐adrenergic signaling on cardiac contractility modulation response in hiPSC‐CMs

**FIGURE 4:**
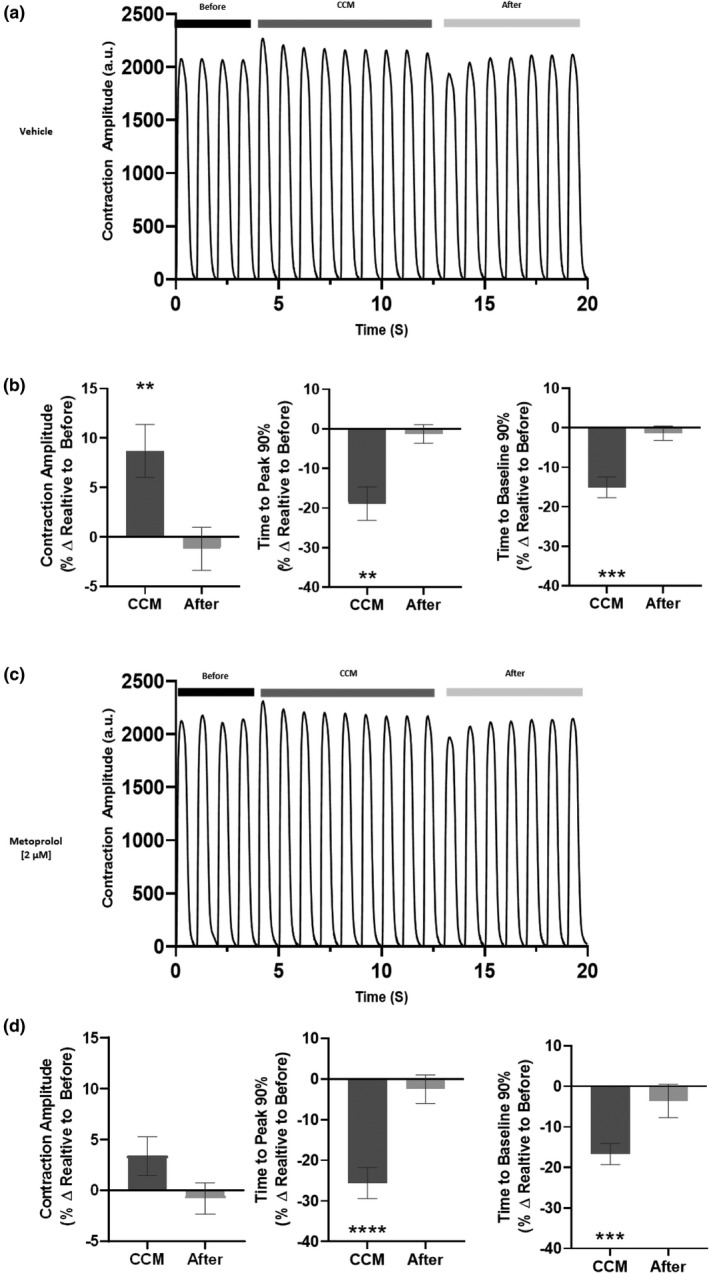
Pharmacological challenge. Representative contraction traces for each group, before (5V), cardiac contractility modulation (CCM) (10V), after (5V), human induced pluripotent stem cell–derived cardiomyocytes were pretreated with (a) Vehicle or (c) metoprolol [2 µM]. (b and d) Summary bar graphs for all beats in each group. Percent change, data are mean ±SEM. *n* = 10 per group. *p < 0.05, **p < 0.01, ***p < 0.001, ****p < 0.0001

To investigate the dependence of β‐adrenergic signaling on acute CCM response in hiPSC‐CMs we assessed the effects of β‐adrenergic blockade by pretreatment with metoprolol [2 µM]. We found pretreatment with metoprolol attenuated the hiPSC‐CM CCM contraction amplitude response but did not inhibit it (Figure 4) suggesting alternative mechanisms may also be contributing to the CCM induce positive inotropic response. Although the CCM induced contractile kinetics effect (time to peak 90% and time to baseline 90%) remained intact independent of β‐adrenergic blockade (Figure 4b).

### 3.6 Cardiac contractility modulation stimulation increases myofilament calcium sensitivity in hiPSC‐CMs

**FIGURE 5:**
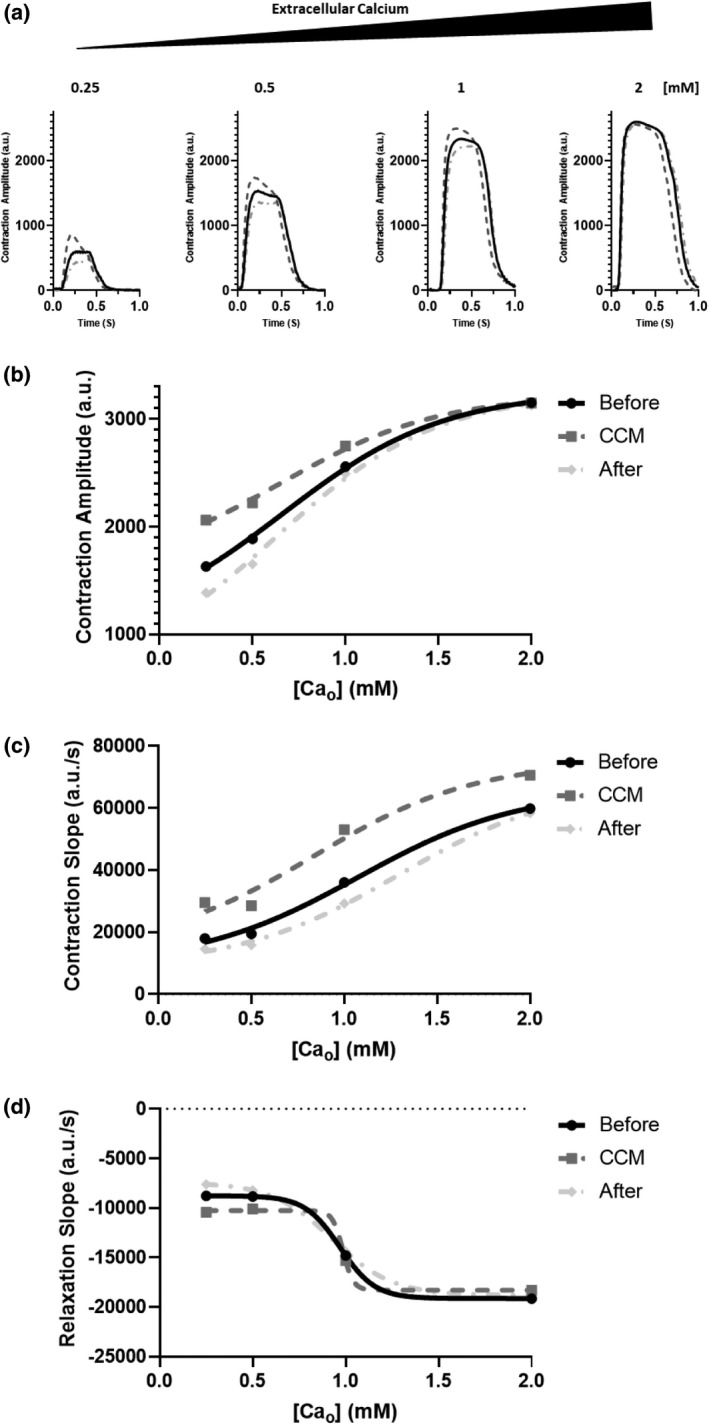
Effect of extracellular calcium modulation on cardiac contractility modulation (CCM) response. (a) Representative contraction traces of immediate effects for each group, before (5V), CCM (10V), after (5V), human induced pluripotent stem cell–derived cardiomyocytes were exposed to increasing concentrations of extracellular calcium [Ca_o_] 0.25–2 mM. (b–d) Transformed data (Sigmoidal), to guide eye, demonstrating the effect of CCM on calcium sensitivity of contractile properties (i.e., amplitude and kinetics) (hill slope = 1.0). *n* = 6 – 8 per group

The effects of CCM were assessed as a function of concentration of extracellular calcium [Ca]_o_ [0.25–2 mM]. CCM stimulation enhanced calcium sensitivity (Figure 5a) as displayed by the leftward shift in the contraction versus calcium concentration curve relative to before CCM stimulation (Figure 5b). This represents increased contraction amplitude at lower concentrations of extracellular calcium suggesting during CCM stimulation the myofilament is more sensitive to calcium. Previous studies have demonstrated the blunted effect of CCM at higher extracellular calcium concentrations (Burkhoff et al., 2001; Brunckhorst et al., 2006) as such we selected [Ca]_o_ [0.5 mM] for contractility, calcium handling, and electrophysiology experiments to enable robust CCM response and maximum assay window. Similarly, L‐type Ca channel blocker, nifedipine [10 nM], significantly enhanced the CCM inotropic response in hiPSC‐CMs (data not shown) further implicating intracellular calcium handling and L‐type calcium channels as contributing CCM mechanisms (Mohri et al., 2002).

## 4 DISCUSSION

### 4.1 Acute cardiac contractility modulation modifies cardiomyocyte function in hiPSC‐CMs

In this study, we establish a robust in vitro method, to quantify the effect of acute CCM stimulation on human cardiomyocytes and improve regulatory decision‐making capabilities. CCM is an intracardiac therapy approved for the treatment of HF with reduced ejection fraction. However, the acute effects of the standard clinical CCM pulse parameters on human cardiomyocytes have not been completely defined. Fluorescence‐ and video‐based imaging was used to quantify the acute effects of clinical CCM parameters on human cardiomyocytes. We demonstrate acute CCM stimulation results in significant increase of contraction, calcium handling, and electrophysiological properties. Moreover, we have for the first time quantified the acute effects of clinical CCM pulses on human cardiomyocytes excitation‐contraction coupling. Importantly, utilization of a multi‐well format enabled a medium‐throughput assay with sufficient replicates to perform meaningful statistical analysis. We previously demonstrated that freshly isolated adult rabbit ventricular cardiomyocytes (rabbit‐CMs) displayed increased contraction and calcium handling properties when stimulated with CCM (Blinova et al., 2014). Conversely, this response was transient and, after the first beat, reduced lower than that of baseline. In this hiPSC‐CM CCM model, we also demonstrate a transient effect for contractility, however, the response remained elevated above baseline after the first beat until CCM was stopped (Table S1). Interestingly, the calcium handling amplitude was significantly reduced, lower than that of baseline −10% (*p* < 0.01) and the calcium‐transient rise time (TRise) was also slower than that of baseline 5% (*p* < 0.05) once the CCM signal was eliminated (Table S2). Although we did not investigate the long‐term consequences post‐CCM, it is foreseeable that these parameters will return to baseline with prolonged recovery time as in the rabbit‐CM model (Blinova et al., 2014). The difference between rabbit‐CMs and hiPSC‐CMs, CCM profile may be a result of species differences. However, it is more likely a result of the hiPSC‐CM model investigated here in monolayer format, as oppose to single cell, representing a syncytium of electrically coupled cells from each cardiac subtype (i.e., ventricular, atrial, and nodal).

Drug‐induced action potential morphology changes are key features for safety hazard assessment. We found CCM stimulation significantly alters the action potential morphology. It is well known that prolongation of the APD, as a result of HERG block, may indicate a possible safety liability (Blinova et al., 2018; Gintant et al., 2020). Although unlikely, significant shortening of the APD may also indicate a potential safety liability when accompanied with action potential triangulation (Hondeghem et al., 2001). Although CCM does significantly shorten the APD, we observed negligible effect on action potential triangulation. Moreover, there are conflicting reports as to the effects of CCM on cardiac electrical activity (Brunckhorst et al., 2006; Winter et al., 2011, 2014; Wood et al., 1969). To the best of our knowledge the effects of CCM stimulation on human QT interval remains unknown. However, one publication demonstrated CCM‐induced attenuation of prolonged QTc interval in a rabbit HF model in vivo (Ning et al., 2020).

β‐adrenergic signaling stimulation has been implicated as a potential mechanism for CCM in vivo (Blinova et al., 2014; Campbell et al., 2020; Winter et al., 2014, 2011). However, CCM is likely induced by a mixed mechanism including sympathetic stimulation through neuronal ganglion and β‐adrenergic signaling pathways as shown by the effects of CCM stimulation on hiPSC‐CM kinetic. The latter result is of critical importance because a β‐adrenergic mechanism does not completely explain the positive inotropic effects observed in the hiPSC‐CM model suggesting alterative mechanisms may be responsible for the positive inotropic effects independent of β‐adrenergic signaling. Additionally, concomitant β‐adrenergic block in the clinical setting does not eliminate the CCM response (Campbell et al., 2020) consistent with our assessment of the β‐adrenergic blocker metoprolol. β‐adrenergic blockers such as metoprolol and propranolol have demonstrated efficacy in uninnervated hiPSC‐CM monocultures suggesting that while the sympathetic stimulation from neurons is absent these cultures may possess an innate level of sympathetic basal tone that can be modulated by β‐adrenergic blockade (Blinova et al., 2018; Grimm et al., 2018; Sirenko et al., 2013; Suzuki et al., 2018).

Pharmacological inotropes traditionally exert their effects by increasing the amount of intracellular calcium available to interact with the myofilament. Calcium enters the cytoplasm predominantly from intracellular calcium stores such as the sarcoplasmic reticulum (Bers, 2002; Hwang et al., 2015) by a mechanism known as calcium‐induced calcium release. Conversely, CCM stimulation has been shown to have more robust effects at lower extracellular calcium concentrations in a rabbit papillary muscle model (Brunckhorst et al., 2006) as such we explored a CCM calcium sensitization hypothesis in which myofilament calcium sensitivity is enhanced resulting in increased contractility at lower levels of extracellular calcium concentration. This mechanism is comparable with calcium sensitizing mutations (Wang et al., 2018) and drugs such as levosimendan and EMD 57033 (Feaster et al., 2015). Previous studies have demonstrated CCM stimulation increased phosphorylation of the inhibitory myofilament protein cardiac troponin I, which regulates myofilament sensitivity (Tschope et al., 2019). Yet, such a phenomenon had not been thoroughly investigated in the context of CCM in a human model.

A calcium‐sensitizing mechanism, mediated by enhanced myofilament calcium sensitivity, partly explains the contractile inotropic effects but does not account for the changes in intracellular calcium transient amplitude induced by CCM. As a result, CCM may also have a direct effect on calcium entry pathways including L‐type Ca channels, ryanodine receptors, and sodium calcium exchanger. This would explain the calcium‐transient amplitude changes induced by CCM and the blunted CCM response at higher extracellular calcium concentrations (i.e., 2 mM) when L‐type Ca channels display increased activation. Detailed pharmacological studies would be needed to establish and quantify the contribution of each of the calcium entry mechanism in the context of the CCM response however this is outside of the scope of this work. Future studies with functionally mature hiPSC‐CM models such as 3D complex co‐culture models (e.g., engineered heart tissue) with intact isoproterenol‐induced positive inotropy and positive force–frequency relationship are predicted to augment the response and shift experimental conditions to more physiologic extracellular Ca concentrations (e.g., ~1.2 mM). Toward this goal, our laboratory is actively working on such experiments.

### 4.2 Comparison of hiPSC‐CM cardiac contractility modulation studies and historical cardiac contractility modulation studies

Previous preclinical CCM studies are difficult to compare as they use a variety of CCM pulse paraments (e.g., duration, monophasic vs. biphasic), model species and location of stimulation site. This results in conflicting reports of CCM effects on cardiomyocyte electrophysiology properties (Burkhoff et al., 2001; Campbell et al., 2020; Winter et al., 2014). Translatability of these results to human and the variability of CCM experimental study pulse parameters makes it difficult to correlate species relevance and compare results between studies, respectively. To mitigate this, we placed a significant emphasis on characterizing the electrical field generated by our system to ensure uniform and consistent CCM stimulation (Figure S3) and also to report important pulse specific details (e.g., V/cm, current, electric field). Despite the discrepancy between studies the overall consensus is that CCM stimulation increases contractility and calcium handling and enhances gene expression without negatively effecting mitochondrial function. One study using a rabbit papillary muscle model demonstrated enhanced contractility and shortened APD in a manner dependent on the CCM pulse parameters (e.g., amplitude polarity). (Brunckhorst et al., 2006) However, this study used nonclinical CCM pulse parameter (i.e., one monophasic pulse). Another study in an isolated rabbit whole heart model demonstrated increased contraction and shorted monophasic APD along with dependance on β‐adrenergic signaling. Likewise, this work used nonclinical CCM pulse parameter (i.e., one biphasic pulse) (Burkhoff et al., 2001). Our work represents the first CCM study using the standard clinical CCM parameters (e.g., two biphasic pulses) (Campbell et al., 2020) in human stem cell–derived cardiomyocytes in vitro.

### 4.3 Study limitations

Our study has several limitations. For example, the CCM pulse parameters tested were selected to best mimic that of the clinical CCM device. Although we demonstrate increased contraction within these parameters, we did not investigate the effects of varying pulse parameters toward the specificity of the observed CCM response (Brunckhorst et al., 2006; Lyon et al., 2013; Winter et al., 2011). Although outside the scope of this work futures studies may investigate such parameters by modulation of CCM delay, duration, waveform (e.g., monophasic), and frequency to determine which combination of pulse parameters are truly responsible for the CCM effects. Likewise, only acute CCM effects were assessed in this study. Chronic CCM effects, consistent with the clinical CCM device, were outside the scope of this study but should include structural, functional, and molecular (e.g., gene expression and mitochondrial) changes induced by prolonged CCM stimulation. For instance, previous studies have demonstrated a reversal of fetal gene expression in HF patients as a result of prolonged CCM stimulation (Abi‐Samra & Gutterman, 2016; Borggrefe et al., 2008; Tschope et al., 2019). Additionally, the commercial hiPSC‐CMs used in this study represents an apparently “healthy” heart model, whereas CCM is indicated for HF patients. This model will be extended to diseased backgrounds including HF. Personalized diseased hiPSC‐CM may display enhanced CCM response relative to healthy hiPSC‐CMs.

HiPSC‐CMs display several features of immature cardiomyocytes including spontaneous beating. In future studies we plan to take advantage of this feature by investigating the effects of CCM at remote locations outside of the ROI, in an effort to elucidate both temporal and spatial effects of chronic CCM stimulation. Moreover, in this study, we used field stimulation to drive hiPSC‐CM pacing. Hence, this does not necessarily capture the exact conditions of the device used in the clinic where the spontaneous beating of the heart is detected by the device then followed by the CCM pulse. Future studies will use an updated CCM pulse generator, which detects spontaneous hiPSC‐CM beating to better mimic the devices used in the clinic.

To ensure robust CCM response, we assess hiPSC‐CMs at submaximal extracellular calcium concentration [0.5 mM], this has a number of implications. (1) [0.5 mM] calcium is comparable with the EC_50_ value for calcium in hiPSC‐CMs (Mannhardt et al., 2016, 2017; Schaaf et al., 2011). (2) Previous studies have demonstrated the blunted effect of CCM at higher extracellular calcium concentration (Mohri et al., 2003) greatly reducing the assay window. (3) Submaximal calcium may be more representative of the clinical device setting, as several studies have implicated impaired calcium handling and reduced L‐type calcium current or expression in the context of HF (Mohri et al., 2002, 2003; Mukherjee et al., 1998). (4) hiPSC‐CMs display relatively mature intracellular calcium handling properties as previously described (Hwang et al., 2015) while previous reports, from the specific donor hiPSC‐CMs used in this study, suggest increased L‐type calcium channel expression relative to human ventricular‐CMs (Blinova et al., 2017, 2019). (5) Submaximal calcium concentration reduces maximum force and contraction amplitude enabling an in vitro reduced ejection fraction “phenotype” (Feaster et al., 2015; Wang et al., 2016). Nevertheless, we cannot exclude the impact of submaximal extracellular calcium on the in vitro CCM response relative to the clinic.

## 5 CONCLUSIONS

This work lays a foundation to improve regulatory decision‐making and support safety and effectiveness studies not only for future CCM devices but also for other cardiac electrophysiology medical devices in general. Here we demonstrate several important findings including (1) hiPSC‐CMs respond to acute clinical CCM stimulation parameters in vitro; (2) CCM stimulation enhances lusitropy independent of its inotropic effect; (3) CCM increases calcium sensitivity at the level of the cardiomyocyte; and (4) CCM stimulation inotropic effects are in part mediated by β‐adrenergic signaling. Moreover, we observed that at higher extracellular calcium concentration, when the CCM‐induced positive inotropy was blunted, the CCM kinetics enhancements remained intact (Figure 5c,d; Figure S2). This further suggests, that CCM may provide a lusitropic benefit to patients independent of the inotropic benefit. This result is of critical importance and may be useful to explore additional patient populations including HF with preserved ejection fraction. Furthermore, it will be interesting to study the effects of additional cardiac electrophysiology devices (e.g., ablation, CRT, and ICD) in this model. Toward that goal, we are actively evaluating cardiac medical devices in a variety of novel in vitro hiPSC models.

## 6 DISCLAIMER

This article reflects the views of the authors and should not be construed to represent the US Food and Drug Administration's views or policies. The mention of commercial products, their sources, or their use in connection with material reported herein is not to be construed as either an actual or implied endorsement of such products by the Department of Health and Human Services.

## DISCLOSURES

The authors declared no competing interest for this work.

## AUTHOR CONTRIBUTIONS

T.F. and K.B. conceived and designed research. T.F., M.C., and A.N. performed experiments and analyzed data. M.C. performed numerical simulation. T.F., M.C., and K.B interpreted the results of experiments. T.F. and K.B. drafted the manuscript; M.C. and A.N. edited and revised the manuscript. T.F., M.C., A.N., and K.B. approved the final version of the manuscript.

## Supporting information



Supplementary Material

## References

[phy215085-bib-0001] Abi‐Samra, F. , & Gutterman, D. (2016). Cardiac contractility modulation: a novel approach for the treatment of heart failure. Heart Failure Reviews, 21(6), 645–660. 10.1007/s10741-016-9571-6. 27394714 PMC5082590

[phy215085-bib-0002] Bers, D. M. (2002). Cardiac excitation‐contraction coupling. Nature, 415(6868), 198–205. 10.1038/415198a. 11805843

[phy215085-bib-0003] Blinova, K. , Dang, Q. , Millard, D. , Smith, G. , Pierson, J. , Guo, L. , Brock, M. , Lu, H. R. , Kraushaar, U. , Zeng, H. , Shi, H. , Zhang, X. , Sawada, K. , Osada, T. , Kanda, Y. , Sekino, Y. , Pang, L. , Feaster, T. K. , Kettenhofen, R. , … Gintant, G. (2018). International multisite study of human‐induced pluripotent stem cell‐derived cardiomyocytes for drug proarrhythmic potential assessment. Cell Reports, 24(13), 3582–3592. 10.1016/j.celrep.2018.08.079. 30257217 PMC6226030

[phy215085-bib-0004] Blinova, K. , Schocken, D. , Patel, D. , Daluwatte, C. , Vicente, J. , Wu, J. C. , & Strauss, D. G. (2019). Clinical trial in a dish: Personalized stem cell‐derived cardiomyocyte assay compared with clinical trial results for two QT‐prolonging drugs. Clinical and Translational Science, 12(6), 687–697. 10.1111/cts.12674. 31328865 PMC6853144

[phy215085-bib-0005] Blinova, K. , Stohlman, J. , Krauthamer, V. , Knapton, A. , Bloomquist, E. , & Gray, R. A. (2014). Acute effects of nonexcitatory electrical stimulation during systole in isolated cardiac myocytes and perfused heart. Physiological Reports, 2(8):e12106. 10.14814/phy2.12106. 25096553 PMC4246583

[phy215085-bib-0006] Blinova, K. , Stohlman, J. , Vicente, J. , Chan, D. , Johannesen, L. , Hortigon‐Vinagre, M. P. , Zamora, V. , Smith, G. , Crumb, W. J. , Pang, L. , Lyn‐Cook, B. , Ross, J. , Brock, M. , Chvatal, S. , Millard, D. , Galeotti, L. , Stockbridge, N. , & Strauss, D. G. (2017). Comprehensive translational assessment of human‐induced pluripotent stem cell derived cardiomyocytes for evaluating drug‐induced arrhythmias. Toxicological Sciences, 155(1), 234–247. 10.1093/toxsci/kfw200. 27701120 PMC6093617

[phy215085-bib-0007] Borggrefe, M. M. , Lawo, T. , Butter, C. , Schmidinger, H. , Lunati, M. , Pieske, B. , Misier, A. R. , Curnis, A. , Bocker, D. , Remppis, A. , Kautzner, J. , Stuhlinger, M. , Leclerq, C. , Taborsky, M. , Frigerio, M. , Parides, M. , Burkhoff, D. , & Hindricks, G. (2008). Randomized, double blind study of non‐excitatory, cardiac contractility modulation electrical impulses for symptomatic heart failure. European Heart Journal, 29(8), 1019–1028. 10.1093/eurheartj/ehn020. 18270213

[phy215085-bib-0008] Brunckhorst, C. B. , Shemer, I. , Mika, Y. , Ben‐Haim, S. A. , & Burkhoff, D. (2006). Cardiac contractility modulation by non‐excitatory currents: studies in isolated cardiac muscle. European Journal of Heart Failure, 8(1), 7–15. 10.1016/j.ejheart.2005.05.011. 16202650

[phy215085-bib-0009] Burkhoff, D. , Shemer, I. , Felzen, B. , Shimizu, J. , Mika, Y. , Dickstein, M. , Prutchi, D. , Darvish, N. , & Ben‐Haim, S. A. (2001). Electric currents applied during the refractory period can modulate cardiac contractility in vitro and in vivo. Heart Failure Reviews, 6(1), 27–34. 10.1023/a:1009851107189. 11248765

[phy215085-bib-0010] Campbell, C. M. , Kahwash, R. , & Abraham, W. T. (2020). Optimizer smart in the treatment of moderate‐to‐severe chronic heart failure. Future Cardiology, 16(1), 13–25. 10.2217/fca-2019-0044. 31825245

[phy215085-bib-0011] FDA.gov . OPTIMIZER Smart System ‐ P180036 2019 [updated 03/21/2019‐07/27/2021]. Available from: https://www.fda.gov/medical‐devices/recently‐approved‐devices/optimizer‐smart‐system‐p180036.

[phy215085-bib-0012] Feaster, T. K. , Cadar, A. G. , Wang, L. , Williams, C. H. , Chun, Y. W. , Hempel, J. , Bloodworth, N. , Merryman, W. D. , Lim, C. C. , Wu, J. C. , Knollmann, B. C. , & Hong, C. C. (2015). Matrigel mattress: A method for the generation of single contracting human‐induced pluripotent stem cell‐derived cardiomyocytes. Circulation Research, 117(12), 995–1000. 10.1161/CIRCRESAHA.115.307580. 26429802 PMC4670592

[phy215085-bib-0013] Gintant, G. , Kaushik, E. P. , Feaster, T. , Stoelzle‐Feix, S. , Kanda, Y. , Osada, T. , Smith, G. , Czysz, K. , Kettenhofen, R. , Lu, H. R. , Cai, B. , Shi, H. , Herron, T. J. , Dang, Q. , Burton, F. , Pang, L. , Traebert, M. , Abassi, Y. , Pierson, J. B. , & Blinova, K. (2020). Repolarization studies using human stem cell‐derived cardiomyocytes: Validation studies and best practice recommendations. Regulatory Toxicology and Pharmacology, 117, 104756. 10.1016/j.yrtph.2020.104756 32822771

[phy215085-bib-0014] Grimm, F. A. , Blanchette, A. , House, J. S. , Ferguson, K. , Hsieh, N. H. , Dalaijamts, C. , Wright, A. A. , Anson, B. , Wright, F. A. , Chiu, W. A. , & Rusyn, I. (2018). A human population‐based organotypic in vitro model for cardiotoxicity screening. Altex, 35(4), 441–452. 10.14573/altex.1805301. 29999168 PMC6231908

[phy215085-bib-0015] Harris, K. , Aylott, M. , Cui, Y. , Louttit, J. B. , McMahon, N. C. , & Sridhar, A. (2013). Comparison of electrophysiological data from human‐induced pluripotent stem cell‐derived cardiomyocytes to functional preclinical safety assays. Toxicological Sciences, 134(2), 412–426. 10.1093/toxsci/kft113. 23690542

[phy215085-bib-0016] Hondeghem, L. M. , Carlsson, L. , & Duker, G. (2001). Instability and triangulation of the action potential predict serious proarrhythmia, but action potential duration prolongation is antiarrhythmic. Circulation, 103(15), 2004–2013. 10.1161/01.cir.103.15.2004. 11306531

[phy215085-bib-0017] Hwang, H. S. , Kryshtal, D. O. , Feaster, T. K. , Sanchez‐Freire, V. , Zhang, J. , Kamp, T. J. , Hong, C. C. , Wu, J. C. , & Knollmann, B. C. (2015). Comparable calcium handling of human iPSC‐derived cardiomyocytes generated by multiple laboratories. Journal of Molecular and Cellular Cardiology, 85, 79–88. 10.1016/j.yjmcc.2015.05.003. 25982839 PMC4530041

[phy215085-bib-0018] Hwang, H. S. , Kryshtal, D. O. , Feaster, T. K. , Sanchez‐Freire, V. , Zhang, J. , Kamp, T. J. , Hong, C. C. , Wu, J. C. , & Knollmann, B. C. (2015). Human induced pluripotent stem cell (hiPSC) derived cardiomyocytes to understand and test cardiac calcium handling: A glass half full. Journal of Molecular and Cellular Cardiology, 89(Pt B), 379–380. 10.1016/j.yjmcc.2015.10.021. 26695095

[phy215085-bib-0019] Jaffe, L. M. , & Morin, D. P. (2014). Cardiac resynchronization therapy: history, present status, and future directions. Ochsner Journal, 14(4), 596–607. PMC4295737 25598725

[phy215085-bib-0020] Lamore, S. D. , Ahlberg, E. , Boyer, S. , Lamb, M. L. , Hortigon‐Vinagre, M. P. , Rodriguez, V. , Smith, G. L. , Sagemark, J. , Carlsson, L. , Bates, S. M. , Choy, A. L. , Stalring, J. , Scott, C. W. , & Peters, M. F. (2017). Deconvoluting kinase inhibitor induced cardiotoxicity. Toxicological Sciences, 158(1), 213–226. 10.1093/toxsci/kfx082. 28453775 PMC5837613

[phy215085-bib-0021] Lyon, A. R. , Samara, M. A. , & Feldman, D. S. (2013). Cardiac contractility modulation therapy in advanced systolic heart failure. Nature Reviews Cardiology, 10(10), 584–598. 10.1038/nrcardio.2013.114. 23939481

[phy215085-bib-0022] Ma, J. , Guo, L. , Fiene, S. J. , Anson, B. D. , Thomson, J. A. , Kamp, T. J. , Kolaja, K. L. , Swanson, B. J. , & January, C. T. (2011). High purity human‐induced pluripotent stem cell‐derived cardiomyocytes: Electrophysiological properties of action potentials and ionic currents. American Journal of Physiology. Heart and Circulatory Physiology, 301(5), H2006–H2017. 10.1152/ajpheart.00694.2011. 21890694 PMC4116414

[phy215085-bib-0023] Mannhardt, I. , Breckwoldt, K. , Letuffe‐Breniere, D. , Schaaf, S. , Schulz, H. , Neuber, C. , Benzin, A. , Werner, T. , Eder, A. , Schulze, T. , Klampe, B. , Christ, T. , Hirt, M. N. , Huebner, N. , Moretti, A. , Eschenhagen, T. , & Hansen, A. (2016). Human engineered heart tissue: Analysis of contractile force. Stem Cell Reports, 7(1), 29–42. 10.1016/j.stemcr.2016.04.011. 27211213 PMC4944531

[phy215085-bib-0024] Mannhardt, I. , Eder, A. , Dumotier, B. , Prondzynski, M. , Kramer, E. , Traebert, M. , Sohren, K. D. , Flenner, F. , Stathopoulou, K. , Lemoine, M. D. , Carrier, L. , Christ, T. , Eschenhagen, T. , & Hansen, A. (2017). Blinded contractility analysis in hipsc‐cardiomyocytes in engineered heart tissue format: Comparison with human atrial trabeculae. Toxicological Sciences, 158(1), 164–175. 10.1093/toxsci/kfx081. 28453742 PMC5837217

[phy215085-bib-0025] Mohri, S. , He, K. L. , Dickstein, M. , Mika, Y. , Shimizu, J. , Shemer, I. , Yi, G. H. , Wang, J. , Ben‐Haim, S. , & Burkhoff, D. (2002). Cardiac contractility modulation by electric currents applied during the refractory period. American Journal of Physiology. Heart and Circulatory Physiology, 282(5), H1642–H1647. 10.1152/ajpheart.00959.2001. 11959626

[phy215085-bib-0026] Mohri, S. , Shimizu, J. , Mika, Y. , Shemer, I. , Wang, J. , Ben‐Haim, S. , & Burkhoff, D. (2003). Electric currents applied during refractory period enhance contractility and systolic calcium in the ferret heart. American Journal of Physiology. Heart and Circulatory Physiology, 284(4), H1119–H1123. 10.1152/ajpheart.00378.2002. 12446280

[phy215085-bib-0027] Mukherjee, R. , Hewett, K. W. , Walker, J. D. , Basler, C. G. , & Spinale, F. G. (1998). Changes in L‐type calcium channel abundance and function during the transition to pacing‐induced congestive heart failure. Cardiovascular Research, 37(2), 432–444. 10.1016/s0008-6363(97)00128-4. 9614498

[phy215085-bib-0028] Ning, B. , Zhang, F. , Song, X. , Hao, Q. , Li, Y. , Li, R. , & Dang, Y. (2020). Cardiac contractility modulation attenuates structural and electrical remodeling in a chronic heart failure rabbit model. Journal of International Medical Research, 48(10), 300060520962910. 10.1177/0300060520962910. PMC7556184 33044118

[phy215085-bib-0029] Saleem, U. , van Meer, B. J. , Katili, P. A. , Yusof, N. A. N. M. , Mannhardt, I. , Garcia, A. K. , Tertoolen, L. , de Korte, T. , Vlaming, M. L. H. , McGlynn, K. , Nebel, J. , Bahinski, A. , Harris, K. , Rossman, E. , Xu, X. , Burton, F. L. , Smith, G. L. , Clements, P. , Mummery, C. L. , … Denning, C. (2020). Blinded, multi‐centre evaluation of drug‐induced changes in contractility using human induced pluripotent stem cell‐derived cardiomyocytes. Toxicological Sciences, 176, 103–123. 10.1093/toxsci/kfaa058. 32421822 PMC7357169

[phy215085-bib-0030] Schaaf, S. , Shibamiya, A. , Mewe, M. , Eder, A. , Stohr, A. , Hirt, M. N. , Rau, T. , Zimmermann, W. H. , Conradi, L. , Eschenhagen, T. , & Hansen, A. (2011). Human engineered heart tissue as a versatile tool in basic research and preclinical toxicology. PLoS One, 6(10), e26397. 10.1371/journal.pone.0026397. 22028871 PMC3197640

[phy215085-bib-0031] Sirenko, O. , Crittenden, C. , Callamaras, N. , Hesley, J. , Chen, Y. W. , Funes, C. , Rusyn, I. , Anson, B. , & Cromwell, E. F. (2013). Multiparameter in vitro assessment of compound effects on cardiomyocyte physiology using iPSC cells. Journal of Biomolecular Screening, 18(1), 39–53. 10.1177/1087057112457590. 22972846

[phy215085-bib-0032] Strauss, D. G. , & Blinova, K. (2017). Clinical trials in a dish. Trends in Pharmacological Sciences, 38(1), 4–7. 10.1016/j.tips.2016.10.009. 27876286 PMC5379998

[phy215085-bib-0033] Summary of Safety and Effectiveness Data (SSED). [updated March 21, 2019; cited 2021]. Available from: https://www.accessdata.fda.gov/cdrh_docs/pdf18/P180036B.pdf

[phy215085-bib-0034] Suzuki, K. , Onishi, T. , Nakada, C. , Takei, S. , Daniels, M. J. , Nakano, M. , Matsuda, T. , & Nagai, T. (2018). Uninterrupted monitoring of drug effects in human‐induced pluripotent stem cell‐derived cardiomyocytes with bioluminescence Ca(2+) microscopy. BMC Research Notes, 11(1), 313. 10.1186/s13104-018-3421-7. 29776438 PMC5960208

[phy215085-bib-0035] Tschope, C. , Kherad, B. , Klein, O. , Lipp, A. , Blaschke, F. , Gutterman, D. , Burkhoff, D. , Hamdani, N. , Spillmann, F. , & Van Linthout, S. (2019). Cardiac contractility modulation: mechanisms of action in heart failure with reduced ejection fraction and beyond. European Journal of Heart Failure, 21(1), 14–22. 10.1002/ejhf.1349. 30485591 PMC6607484

[phy215085-bib-0036] Wang, L. , Kim, K. , Parikh, S. , Cadar, A. G. , Bersell, K. R. , He, H. , Pinto, J. R. , Kryshtal, D. O. , & Knollmann, B. C. (2018). Hypertrophic cardiomyopathy‐linked mutation in troponin T causes myofibrillar disarray and pro‐arrhythmic action potential changes in human iPSC cardiomyocytes. Journal of Molecular and Cellular Cardiology, 114, 320–327. 10.1016/j.yjmcc.2017.12.002. 29217433 PMC5800960

[phy215085-bib-0037] Wang, Y. , Ma, H. , Hao, X. , Yang, J. , Chen, Q. , Lu, L. , & Zhang, R. (2016). Low serum calcium is associated with left ventricular systolic dysfunction in a Chinese population with coronary artery disease. Scientific Reports, 6, 22283. 10.1038/srep22283. 26924008 PMC4770278

[phy215085-bib-0038] Winter, J. , Brack, K. E. , Coote, J. H. , & Ng, G. A. (2014). Cardiac contractility modulation increases action potential duration dispersion and decreases ventricular fibrillation threshold via beta1‐adrenoceptor activation in the crystalloid perfused normal rabbit heart. International Journal of Cardiology, 172(1), 144–154. 10.1016/j.ijcard.2013.12.184. 24456882 PMC3978661

[phy215085-bib-0039] Winter, J. , Brack, K. E. , & Ng, G. A. (2011). The acute inotropic effects of cardiac contractility modulation (CCM) are associated with action potential duration shortening and mediated by beta1‐adrenoceptor signalling. Journal of Molecular and Cellular Cardiology, 51(2), 252–262. 10.1016/j.yjmcc.2011.04.010. 21557948 PMC3176912

[phy215085-bib-0040] Wood, E. H. , Heppner, R. L. , & Weidmann, S. (1969). Inotropic effects of electric currents. I. Positive and negative effects of constant electric currents or current pulses applied during cardiac action potentials. II. Hypotheses: calcium movements, excitation‐contraction coupling and inotropic effects. Circulation Research, 24(3), 409–445. 10.1161/01.res.24.3.409. 5766519

