# Supplementary material for: Acute effects of cardiac contractility modulation on human induced pluripotent stem cell–derived cardiomyocytes
Source: Physiol Rep. 2021 Nov 3;9(21):e15085. doi: 10.14814/phy2.15085 (PMC8564440; doi:10.14814/phy2.15085)

**Supplemental Table 1.** Contractile Properties. Percent change relative to before (5V), data are mean ± SEM for all beats in each group CCM (10V), and After (5V). n = 23. **P* < 0.05, ***P* < 0.01, ****P* < 0.001, *****P* < 0.0001.

**Supplemental Table 2.** Calcium Handling Properties. Percent change relative to before (5V), data are mean ± SEM for all beats in each group, CCM (10V), and After (5V). n = 13. **P* < 0.05, ***P* < 0.01, ****P* < 0.001, *****P* < 0.0001.

**Supplemental Table 3.** Electrophysiological Properties. Percent change relative to before (5V), data are mean ± SEM for all beats in each group; CCM (10V), and After (5V). n = 12. **P* < 0.05, ***P* < 0.01, ****P* < 0.001, *****P* < 0.0001.

**Supplemental Table 4.** Baseline hiPSC-CM Properties**.** Absolute values of baseline (i.e., Before, 5V) cardiomyocyte excitation-contraction coupling properties for action potential (i.e., electrophysiology), calcium handing and contraction, data are mean ± SEM; n = 5 – 23. N/A = not applicable.

**Supplemental Figure 1.** Schematic of human in vitro CCM Model. A: hiPSC-CMs are pre-plated in monolayer format on gelatin (0.1%) coated 6-well plates. B: After 2 – 28 days in culture hiPSC-CMs are dissociated and prepared for plating on Matrigel mattress substrate. C: Isolated hiPSC-CMs are plated at high density on Matrigel mattress arrayed in 48-well format (left) and assayed in [0.5 mM] [Ca]_0_ Tyrode solution (right). D: Commercial pulse generator and standard clinical CCM pulse parameters (right) are used stimulate hiPSC-CMs, cardiac function is assessed by video and florescence measurements (left). E: Representative contraction recording before CCM (5V), CCM (10V) and after (5V).

**Supplemental Figure 2.** Percent Change for the Effect of Extracellular Calcium Modulation on CCM Response. hiPSC-CMs were exposed to increasing concentrations of extracellular Ca [Ca_o_] 0.25 – 2 mM. Summary bar graphs of immediate effects. n = 6 - 8 per group. Transformed data from figure 5. **P* < 0.05, ***P* < 0.01, ****P* < 0.001, *****P* < 0.0001.

**Supplemental Figure 3**. Electric Field Numerical Modeling. A: Prospective and top view of the geometry of the platinum electrodes inserted in a glass bottom well. B: Electric field intensity in the YZ plane perpendicular to the electrodes for 1 V applied. For quantitative analysis, the values of the |E| field were extracted in the region of interest (ROI). C:|E| field along the y axis; the ROI is highlighted by a rectangle. D: Scaling table to convert the |E| field in the ROI obtained at 1 V applied to significant experimental values, 5 V and 10 V, respectively.

**Supplemental Figure 4.** Effect of Voltage Amplitude on CCM Response. A: Contraction traces for each group, Before (5V), CCM (0 - 15V), After (5V), hiPSC-CMs were exposed to increasing CCM pulse amplitude (0 – 15) Volts. [Ca_o_] 0.5 mM. B: Transformed data demonstrating the effect of CCM pulse amplitude on contraction amplitude During CCM (Hill slope = 8.0). n = 1. EV_50_ = Effective Voltage 50%.

**Supplemental Figure 5.** Effect of 0V CCM on hiPSC-CM Contractility. A: Contraction traces for each group, Before (5V), CCM (0V), After (5V). [Ca_o_] 0.5 mM.

**Supplemental Video**. hiPSC-CMs on Matrigel Mattress. Matrigel Mattress in one well of 48-well glass bottom plate 4x. hiPSC-CMs form monolayer morphology and robust contraction at ~ 24 hours post plating. White arrow indicated edge of Matrigel mattress. Scale bar, 1 mm.

| **Supplemental Table 1. Contractile Properties** | | |
| --- | --- | --- |
| **Parameter** | **CCM** | **After** |
| **Amplitude** | **16 ± 4%**** | **4 ± 5%** |
| **Time to Peak 50%** | **-20 ± 9%*** | **7 ± 5%** |
| **Time to Peak 90%** | **-22 ± 8%*** | **6 ± 5%** |
| **Time to Baseline 50%** | **-8 ± 5%** | **4 ± 4%** |
| **Time to Baseline 90%** | **-12 ± 6%*** | **5 ± 5%** |
| **Contraction Duration 10%** | **-13 ± 6%** | **3 ± 5%** |
| **Contraction Duration 50%** | **-6 ± 5 %** | **3 ± 5%** |
| **Contraction Duration 90%** | **0 ± 5%** | **3 ± 4%** |
| **N** | **23** | **23** |

| **Supplemental Table 2. Calcium Handling Properties** | | |
| --- | --- | --- |
| **Parameter** | **CCM** | **After** |
| **Amplitude** | **13 ± 5%*** | **-10 ± 2%**** |
| **Time to peak** | **-22 ± 3%****** | **-1 ± 3%** |
| **Ca Rise Time** | **-33 ± 3%****** | **5 ± 2%*** |
| **Ca Duration 50%** | **-10 ± 2%***** | **0 ± 1%** |
| **Ca Duration 90%** | **-2 ± 1%** | **1 ± 1%** |
| **N** | **13** | **13** |

| **Supplemental Table 3. Electrophysiological Properties** | | |
| --- | --- | --- |
| **Parameter** | **CCM** | **After** |
| **TRise** | **-13 ± 5%*** | **-6 ± 17%** |
| **APD50** | **-8 ± 2%**** | **18 ± 6%** |
| **APD75** | **-9 ± 1%****** | **18 ± 4%** |
| **APD90** | **-10 ± 1%****** | **17 ± 5%** |
| **n** | **12** | **12** |

| **Supplemental Table 4. Baseline hiPSC-CM Properties** | | | | |
| --- | --- | --- | --- | --- |
| **Parameter** | **Action Potential** | **Calcium** | | **Contraction** |
| **Amplitude (a.u.)** | **N/A** | **0.20 ± 0.03** | **1882.5 ± 187.2** | |
| **Time to Peak 50% (ms)** | **N/A** | **N/A** | **166.4 ± 15.5** | |
| **Time to Peak 90% (ms)** | **N/A** | **272.6 ± 15.0^#^** | **245.4 ± 18.1** | |
| **TRise (ms)** | **70.0 ± 23.9** | **100.5 ±7.2** | **N/A** | |
| **Contraction / CaT (Up), Slope (a.u./s)** | **N/A** | **22104 ± 5517** | **20088.6 ± 1920.0** | |
| **Time to Baseline 50% (ms)** | **N/A** | **N/A** | **666.2 ± 29.0** | |
| **Time to Baseline 90% (ms)** | **N/A** | **N/A** | **807.5 ± 21.1** | |
| **Relaxation or CaT (Down), Slope (a.u./s)** | **NA** | **-8926 ± 2144** | **-9055.8 ± 843.8** | |
| **Duration 10% (ms)** | **N/A** | **N/A** | **375.4 ± 21.1** | |
| **Duration 50% (ms)** | **519.7 ± 24.2** | **517.1 ± 17.0** | **500.0 ± 19.3** | |
| **Duration 75% (ms)** | **581.1 ± 24.2** | **N/A** | **N/A** | |
| **Duration 90% (ms)** | **613.7 ± 19.4** | **676.0 ± 10.3** | **561.8 ± 13.0** | |
| **Beat Rate, Spontaneous (BPM)** | **N/A** | **N/A** | **33.3 ± 6.4** | |
| **Interval, Spontaneous (ms)** | **N/A** | **N/A** | **2353.2 ± 472.4** | |
| **Interval, Paced 1 Hz (ms)** | **N/A** | **N/A** | **998.18 ± 2.7** | |
| **Beat Rate, Paced 1 Hz (BPM)** | **60** | **60** | **60** | |
| **# = Time to 100%** |  |  |  | |


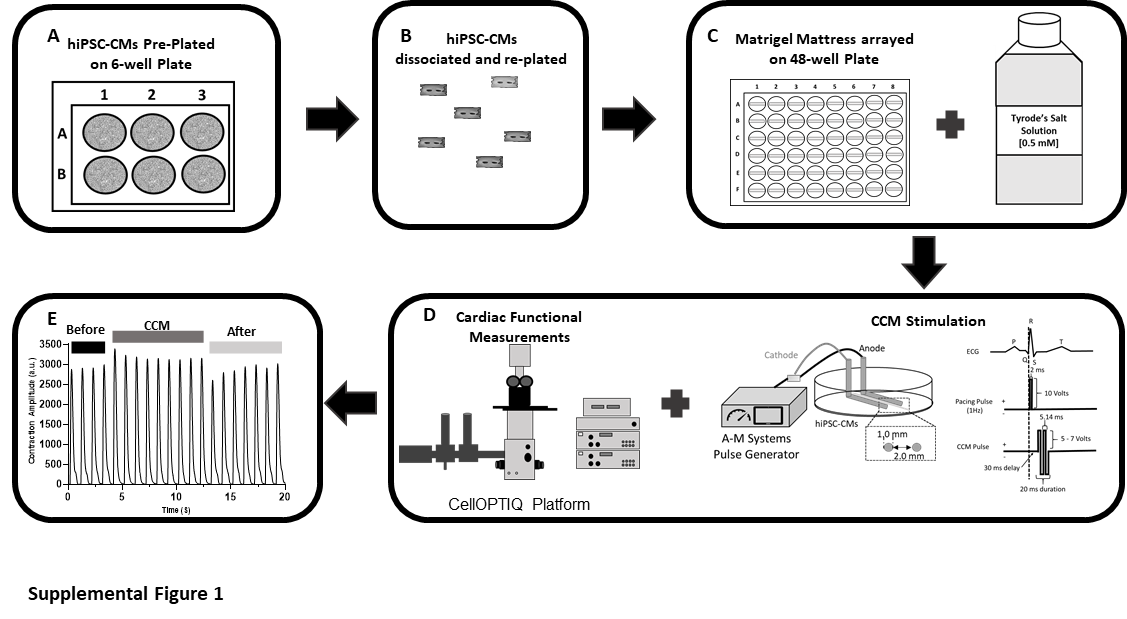


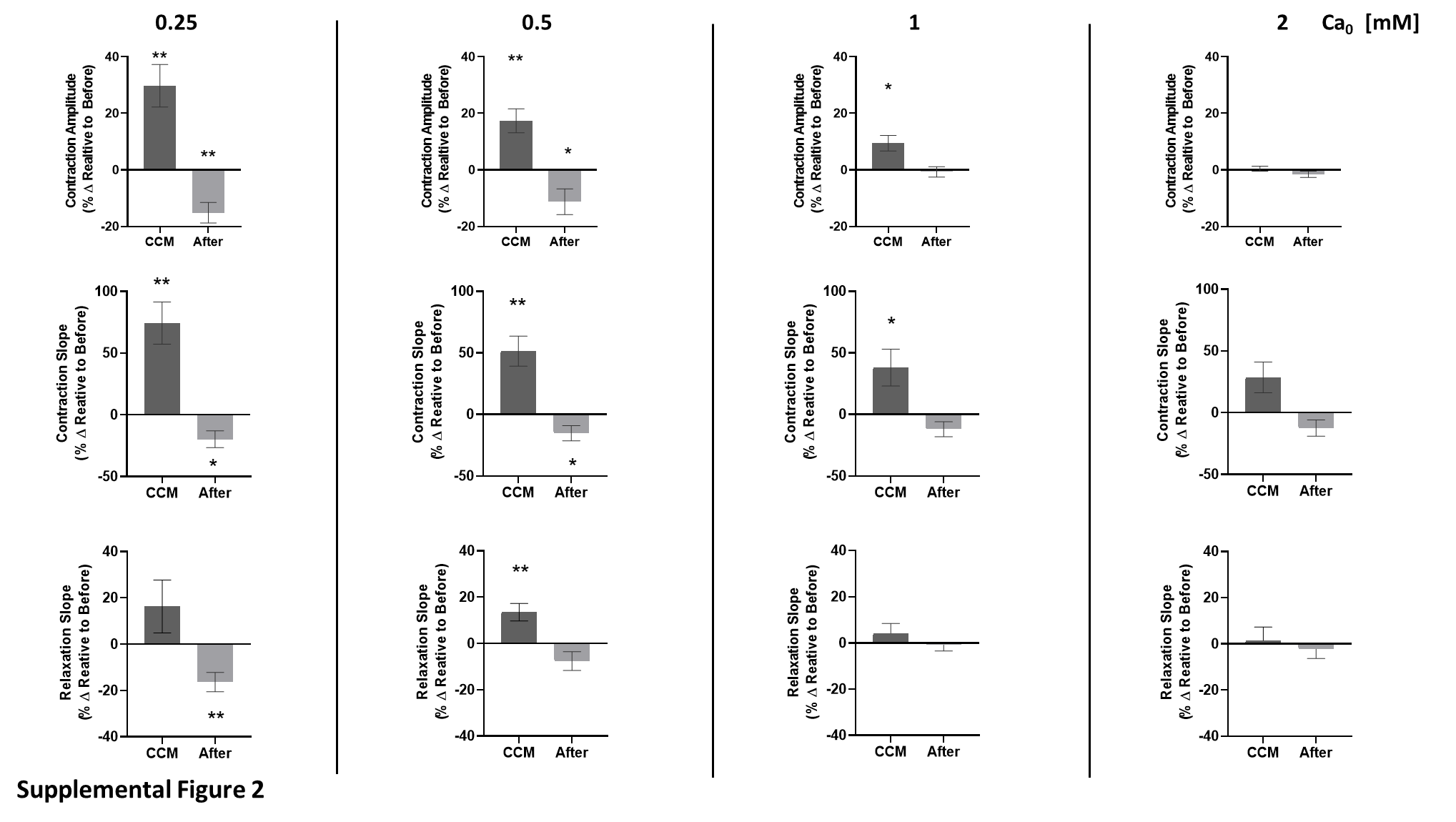


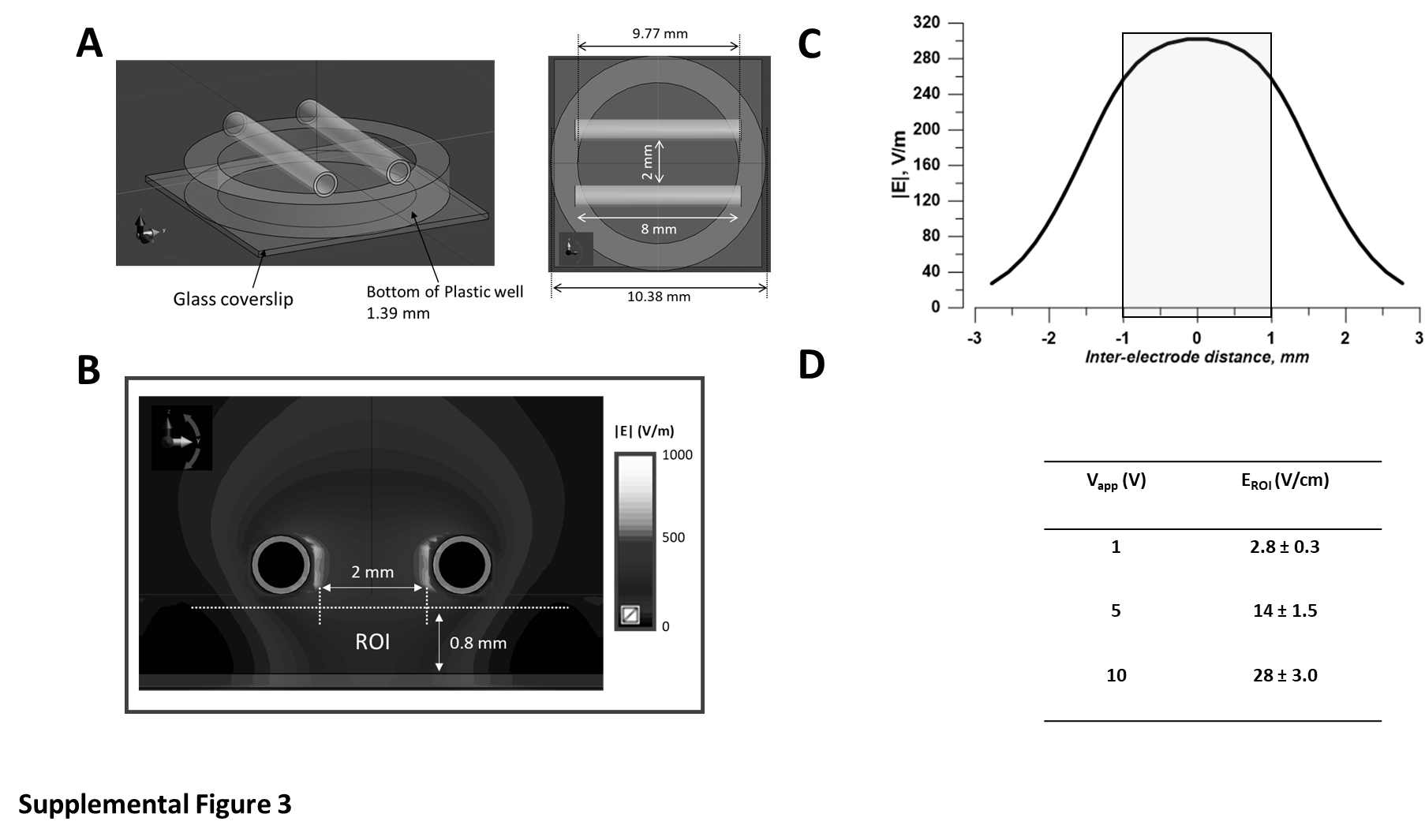


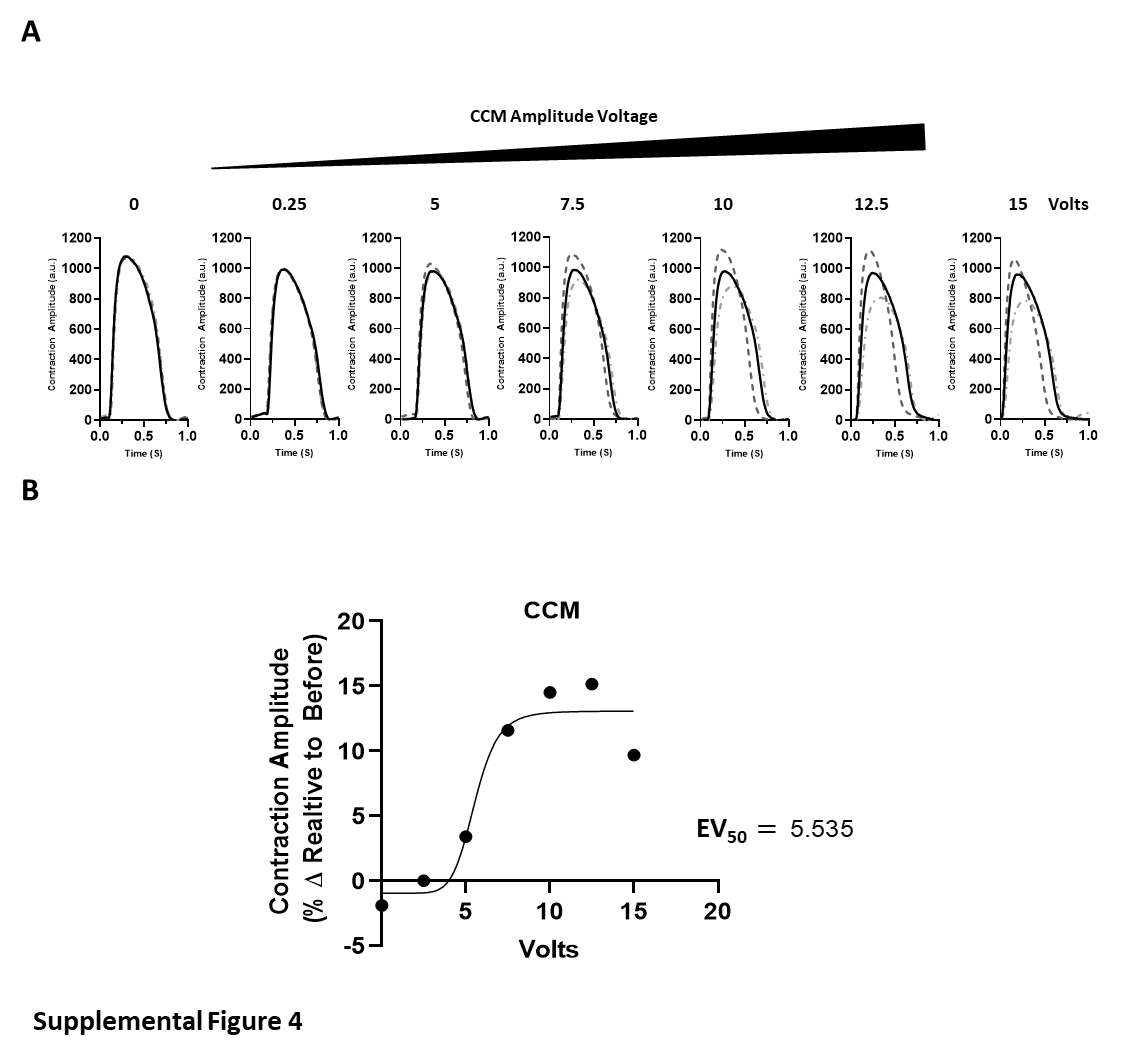


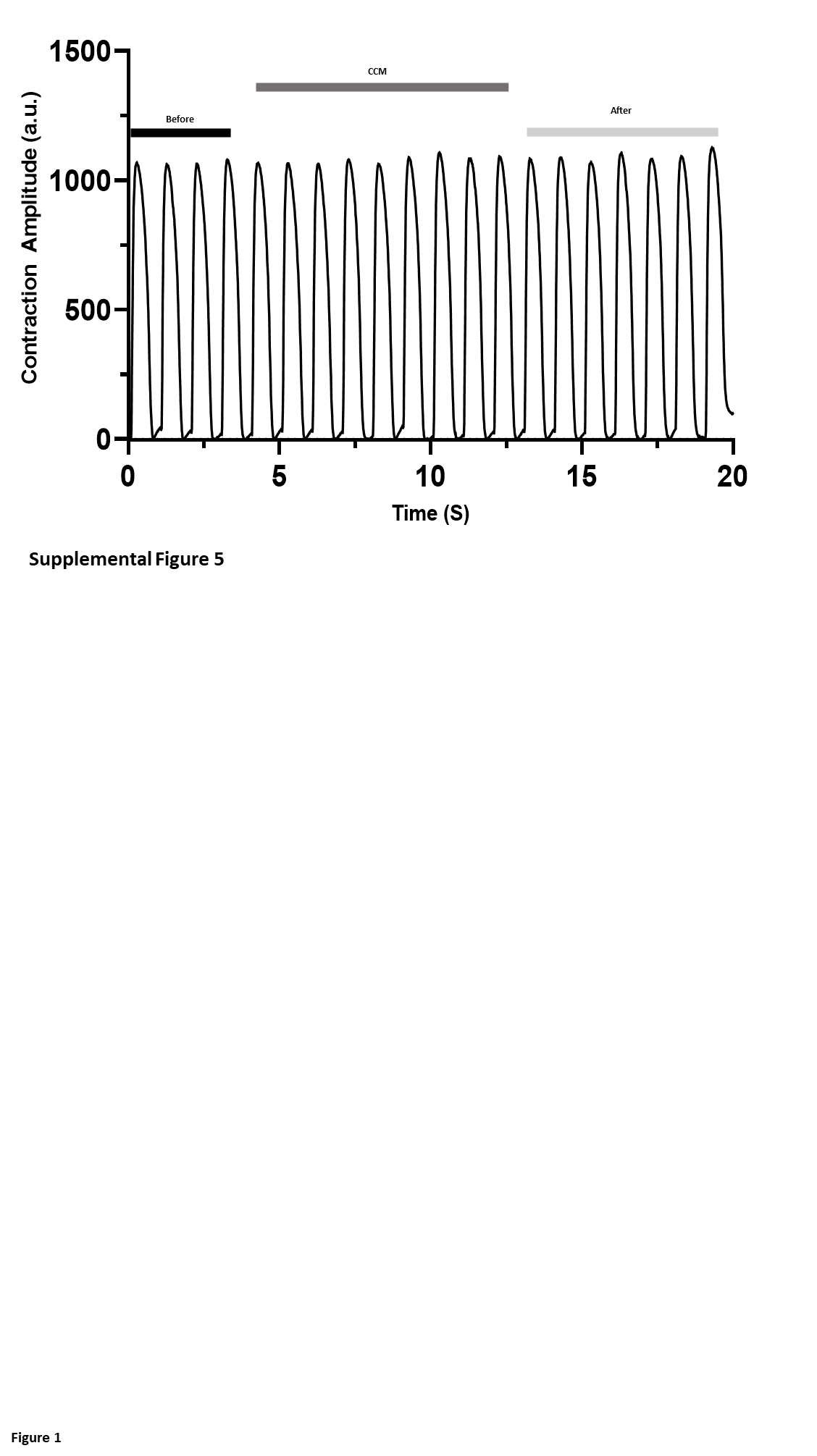


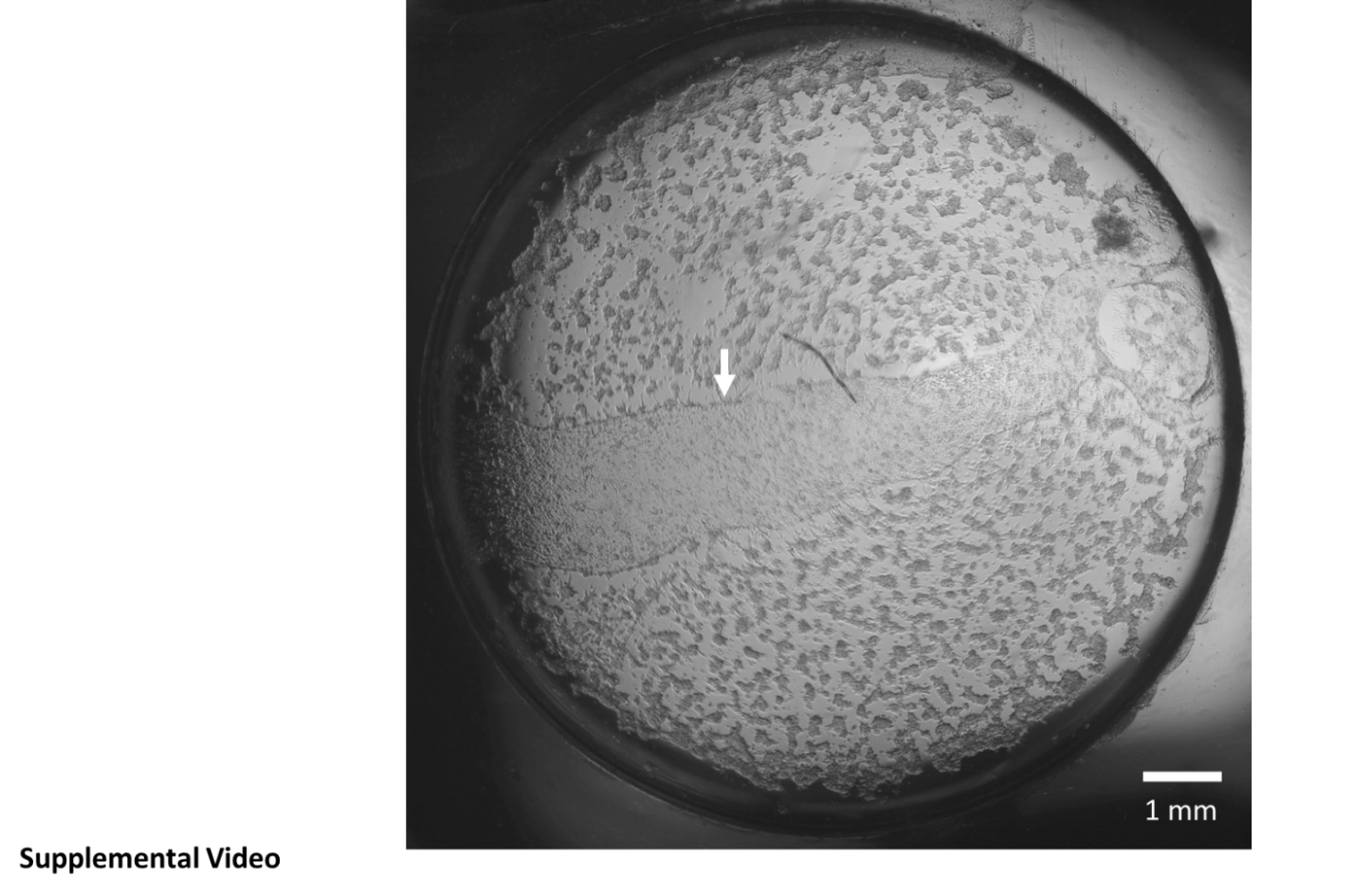

Supplement: Supplementary file 1 — Supplementary Material [file PHY2-9-e15085-s002.docx]
